# Identification of miRNAs as diagnostic and prognostic markers in hepatocellular carcinoma

**DOI:** 10.18632/aging.202606

**Published:** 2021-02-22

**Authors:** Hao Liang, Mingxing Xu, Zhiyong Xiong, Kunpeng Hu, Jiarui Yang, Mingbo Cao, Zhaozhong Zhong, Zhicheng Yao, Meihai Deng, Bo Liu

**Affiliations:** 1 Department of General Surgery, The Third Affiliated Hospital, Sun Yat-sen University, Guangzhou, China; 2 Department of Hepatobiliary Surgery, The Third Affiliated Hospital, Sun Yat-sen University, Guangzhou, China

**Keywords:** hepatocellular carcinoma, miRNAs, bioinformatics, GEO database, biomarker

## Abstract

The development of high-throughput technologies has yielded a large amount of data from molecular and epigenetic analysis that could be useful for identifying novel biomarkers of cancers. We analyzed Gene Expression Omnibus (GEO) DataSet micro–ribonucleic acid (miRNA) profiling datasets to identify miRNAs that could have value as diagnostic and prognostic biomarkers in hepatocellular carcinoma (HCC). We adopted several computing methods to identify the functional roles of these miRNAs. Ultimately, via integrated analysis of three GEO DataSets, three differential miRNAs were identified as valuable markers in HCC. Combining the results of receiver operating characteristic (ROC) analyses and Kaplan–Meier Plotter (KM) survival analyses, we identified hsa-let-7e as a novel potential biomarker for HCC diagnosis and prognosis. Then, we found via quantitative reverse-transcription polymerase chain reaction (RT-qPCR) that let-7e was upregulated in HCC tissues and that such upregulation was significantly associated with poor prognosis in HCC. The results of functional analysis indicated that upregulated let-7e promoted tumor cell growth and proliferation. Additionally, via mechanistic analysis, we found that let-7e could regulate mitochondrial apoptosis and autophagy to adjust and control cancer cell proliferation. Therefore, the integrated results of our bioinformatics analyses of both clinical and experimental data showed that let-7e was a novel biomarker for HCC diagnosis and prognosis and might be a new treatment target.

## INTRODUCTION

Hepatocellular carcinoma (HCC) is the sixth most commonly diagnosed cancer and the fourth leading cause of cancer deaths worldwide [1]. Treatment for HCC has been developed over many years, and includes approaches such as curative resection, ablation, liver transplantation, radiotherapy, cancer pharmacological treatments and transarterial chemoembolization (TACE). However, clinical outcomes of HCC patients remain unsatisfactory due to high recurrence and metastasis rates [2–7]. The probability of postoperative recurrence remains high in patients who undergo surgery, with a 5-year recurrence rate of >70%, and which usually increases within the first 2 years [8]. Meanwhile, the 5-year survival rate is only 30–40% [9]. The complexity of underlying molecular mechanisms in HCC, which could involve genetic mutations, epigenetic alterations and lack of reliable gene signatures [10, 11], leads to poor curative effects. Therefore, understanding the specific mechanisms of HCC pathogenesis and finding therapeutic strategies are urgently needed for better prediction of prognoses and improved treatment in HCC patients.

Micro–ribonucleic acids (miRNAs) are short, single-stranded, noncoding RNAs (ncRNAs) 19–25 nucleotides long that bind to the 3′-untranslated region (UTR) of messenger RNAs (mRNAs), resulting in degradation of the target mRNA molecules or translational inhibition [12]. MiRNAs are multifunctional molecules participating in cell development, differentiation and aging [13–15]. However, the miRNA profiles of malignancies are significantly different from those of normal tissues, making them potentially appealing biomarkers in the diagnosis, treatment and prognosis of various cancers [12, 16, 17]. Recently, the role of miRNAs in tumorigenesis and tumor progression has attracted much attention. A growing amount of evidence has proven the vital role of dysregulated miRNAs in cancer diagnosis and prognosis. Certain miRNAs are significantly correlated with the presence of tumors, even in the early stages, or with worse prognosis [18, 19]. In this study, we focused on finding miRNAs that could have value as diagnostic and prognostic biomarkers in HCC.

With the development of high-throughput technologies, a large amount of data has been generated from molecular and epigenetic analyses. The relevant databases could be used to identify novel biomarkers of cancers via computational approaches [20]. Herein, we analyzed Gene Expression Omnibus (GEO) DataSet miRNA profiling datasets to identify miRNAs with potential diagnostic- and prognostic-biomarker value in HCC. Then, we verified the prognostic values of these miRNAs using the Kaplan–Meier (KM) Plotter (http://www.kmplot.com/analysis/). Further analyses of these miRNAs included Gene Ontology (GO) analysis and Kyoto Encyclopedia of Genes and Genomes (KEGG) signaling pathway analysis. Finally, we identified the most valuable miRNA, hsa-let-7e. Note, however, that bioinformatics data analyses based on the abovementioned databases often produce conflicting results [21]. Therefore, we performed studies to identify the clinical value, biological function (BF) and molecular mechanism (MM) of let-7e. Differences in let-7e expression in HCC tissues were verified and their clinical relevance analyzed. Finally, via *in vitro* experiments, we demonstrated the potential BF and MM of let-7e in HCC. Our results suggested that let-7e might be a novel biomarker for diagnosis, treatment and prognosis in HCC.

## RESULTS

### Identification of significantly dysregulated miRNAs and their diagnostic and prognostic values in HCC tissues

**Figure 1:**
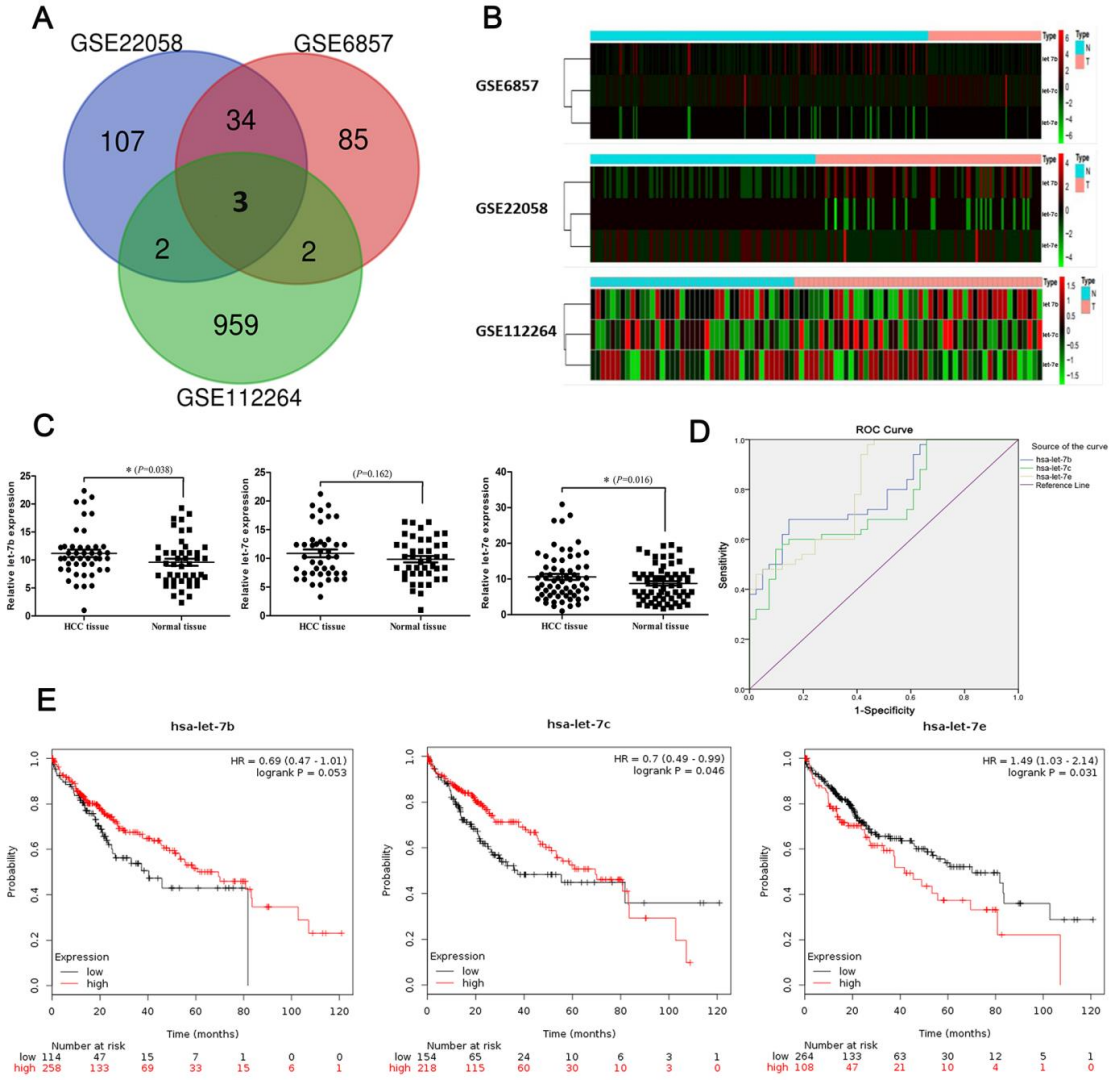
**Identification of significantly dysregulated miRNAs in HCC.** (**A**) Heatmaps of expression profiles of distinct miRNAs in three GEO DataSets (GSE6857, GSE22058 and GSE12264). (**B**) Venn diagram showing number of dysregulated miRNAs found via integrated analysis of the three datasets. (**C**) Expression of let-7b (45 cases; *P=*0.038), let-7c (43 cases; *P=*0.162) and let-7e (63 cases; *P=*0.016) in HCC tissues and matched normal tissues as shown by RT-qPCR. (**D**) ROCs of diagnostic value of the three miRNAs in HCC were computed based on GEO DataSet GSE12264. (**E**) The prognostic roles of the three miRNAs were evaluated via survival analyses based on KM Plotter datasets. Differences in survival rate were compared using a log-rank test.

We performed a comprehensive differential analysis of miRNA expression based on three GEO DataSets (GSE6857, GSE22058 and GSE12264) in order to identify dysregulated miRNAs in HCC tissue compared with non-tumor normal controls (NCs). Datasets GSE6857, GSE22058 and GSE12264, respectively, contained 124, 146 and 966 miRNAs. By merging the three datasets, we were able to identify three consistently dysregulated miRNAs (hsa-let-7b, hsa-let-7c and hsa-let-7e) in HCC tissue versus NCs (Figure 1A). The expression profiles of these distinct miRNAs are shown in the heatmaps in Figure 1B. heatmaps. Considering the widely differing expression levels of hsa-let-7b, hsa-let-7c and/or hsa-let-7e between cancerous and non-tumor tissues, we selected them for further investigation of whether, when dysregulated, they could serve as diagnostic and prognostic markers in HCC.

Next, we used quantitative reverse-transcription polymerase chain reaction (RT-qPCR) to measure differences in these three miRNAs’ expression levels between HCC tissue specimens and matched non-tumor tissue specimens. The results showed that let-7b (*P=*0.038; 45 pairs of specimens) and let-7e (*P=*0.016; 63 pairs) were upregulated in HCC tissue compared with NCs. However, expression of let-7c (*P=*0.162; 43 pairs) did not differ between HCC and normal tissues (Figure 1C).

To further elucidate the diagnostic roles played by the three miRNAs in HCC, we computed receiver operating characteristic (ROC) curves of their diagnostic values in HCC based on the dataset GSE12264, and then we compared the areas under the curve (AUCs) of the ROC curves among the miRNAs (Figure 1D). The results indicated that expression levels of let-7b (AUC, 0.791; 95% CI, 0.700–0.881), let-7c (AUC, 0.740; 95% CI, 0.639–0.841) and let-7e (AUC, 0.810; 95% CI, 0.721–0.898) were significantly higher in HCC tissues than in NCs (Table 1). Then, to the evaluate three miRNAs’ prognostic capabilities, we analyzed patient survival rates using KM Plotter datasets and compared differences in survival rate using a log-rank test. We found that expression levels of let-7c (HR, 0.70; 95%CI, 0.49–0.99, *P=*0.046) and let-7e (HR, 1.49; 95%CI, 1.03–2.14, *P=*0.031) were associated with survival rate in HCC patients. However, expression of let-7b (HR, 0.69; 95%CI, 0.47–1.01; *P=*0.053) had no statistically significant association with survival rate in this analysis (Figure 1E).

**Table 1:** The ROC test results of 3 candidate miRNAs.

**miRNA**	**Area**	***P* value**	**95% Confidence interval**	
			**Lower bound**	**Upper bound**
hsa-let-7b	0.791	*p*<0.001	0.700	0.881
hsa-let-7c	0.740	*p*<0.001	0.639	0.841
hsa-let-7e	0.810	*p*<0.001	0.721	0.898

ROC: Receiver operating characteristics.

### Functional enrichment analysis

**Figure 2:**
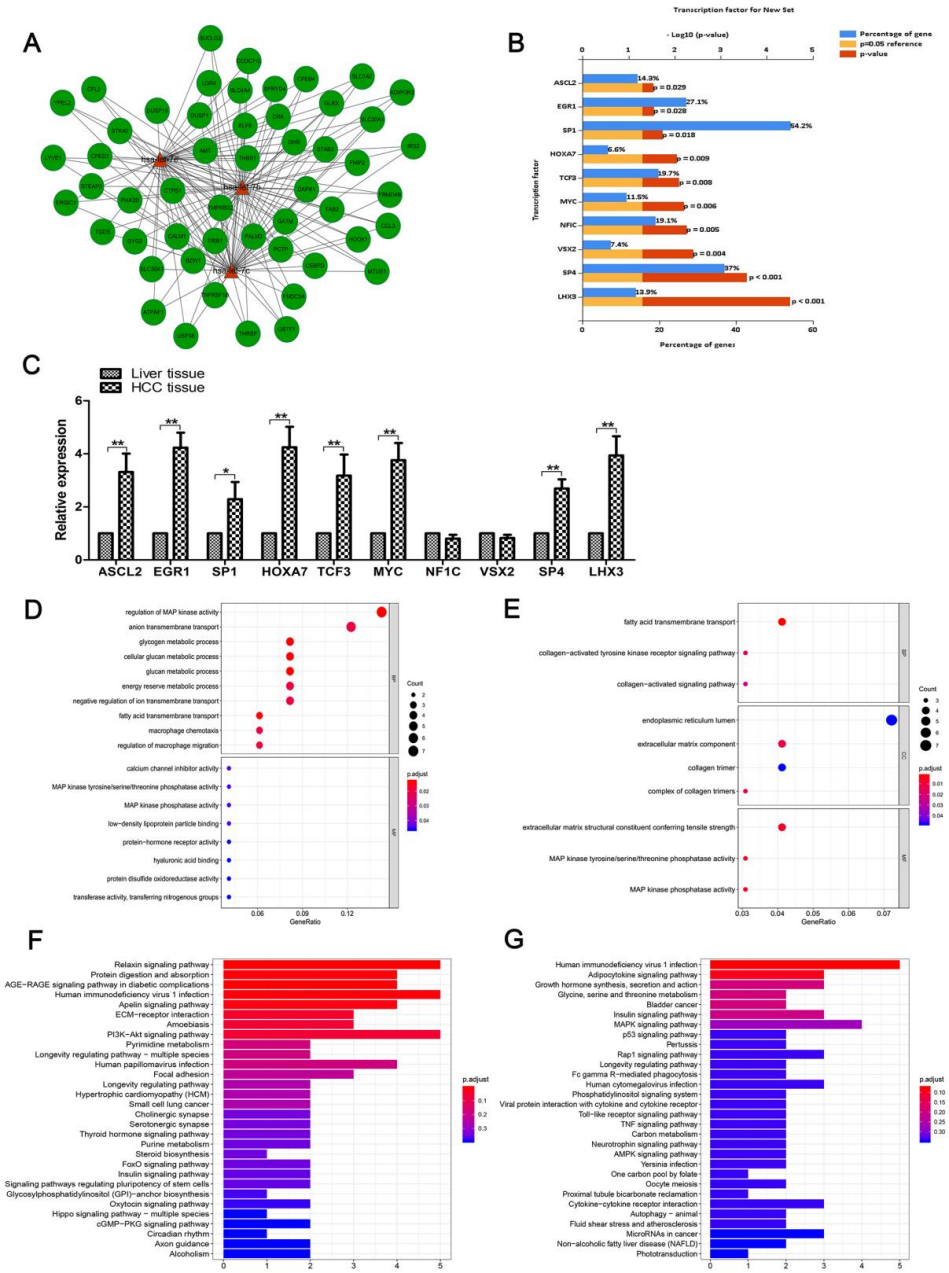
**Functional enrichment analysis.** (**A**) The network of cross-linked genes of let-7b, let-7c and let-7e was constructed of these three miRNAs and 52 genes. (**B**) Top 10 relevant enriched TFs of let-7b, let-7c and let-7e were analyzed, including ASCL2, EGR1, SP1, HOXA7, TCF3, MYC, NF1C, VSX2, SP4 and LHX3. (**C**) Expression of the aforementioned 10 TFs in HCC tissues and matched normal tissues as shown by RT-qPCR. (**D**) GO analysis of genes negatively correlated with the three miRNAs. (**E**) GO analysis of genes positively correlated with these miRNAs. (**F**) KEGG pathway enrichment analysis was conducted to identify the potential pathways of genes positively correlated with the miRNAs. (**G**) KEGG pathway enrichment analysis of genes negatively correlated with the miRNAs.

Using a functional-enrichment analysis tool (FunRich; http://www.funrich.org), we analyzed the most relevant neighboring and cross-linked target genes of the three miRNAs. The result was a network (Figure 2A), which was composed of these miRNAs and of 52 genes. Next, we analyzed the relevant transcription factors (TFs) of let-7b, let-7c and let-7e using FunRich. The top-10 relevant enriched TFs were Achaete–Scute family basic helix-loop-helix (BHLH) transcription factor 2 (ASCL2), early growth response 1 (EGR1), specificity protein 1 (SP1), homeobox A7 (HOXA7), transcription factor 3 (TCF3), myelocytomatosis oncogene (MYC), neurofibromin 1C (NF1C), visual system homeobox 2 (VSX2), SP4 and LIM homeobox 3 (LHX3) (Figure 2B). Then, we measured their expression levels in HCC specimens and matched non-tumor specimens using RT-qPCR. Results are shown in Figure 2C.

The target gene modules in the network were often enriched with diverse specific functions that had biological significance. In order to identify the BFs of the three miRNAs’ target genes, we performed GO analysis based on information from selected genes. Because miRNAs can promote degradation of miRNAs by binding to mRNA, it is helpful to distinguish up and downregulated mRNA groups for further research and analysis into the mechanism of miRNAs. First, we included the genes negatively correlated with the miRNAs in our analysis. The results showed that these genes were enriched in the regulation of mitogen-activated protein kinase (MAPK) activity and anion transmembrane transport in biological processes (BP) (Figure 2D). Then, we included the genes positively correlated with the miRNAs in our GO analysis. Our results indicated that three GO terms of BP, two GO terms of Cellular Component (CC) and three GO terms of Molecular Function (MF) were enriched; we identified these as significant (Figure 2E). In addition, we performed KEGG pathway enrichment analysis to identify potential pathways of the genes positively (Figure 2F) and negatively (Figure 2G) correlated with miRNAs.

### High expression of let-7e was associated with poor clinical outcomes in HCC

The above results showed that let-7e could be the most significant biomarker for diagnosis and prognosis in HCC; it was upregulated in primary HCC tissues compared with adjacent normal tissues in 63 patients. Next, we explored the relationship between let-7e expression and clinicopathological parameters, using median expression level as the cutoff value between the high- and low-expression groups. Results are summarized in Table 2. High let-7e expression was significantly associated with poor tumor differentiation (*P=*0.014), larger tumor size (*P=*0.029) and venous invasion (*P=*0.031).

**Table 2:** Association between let-7e expression and clinicopathological parameters in HCC.

**Variable**	**Patients (n) N=63**	**Let-7e expression (qPCR)**		***P* value**
		**Low(n=31)**	**High(n=32)**	
*Age*				0.701
<60	30	14	16	
≥60	33	17	16	
*Gender*				0.260
Male	35	15	20	
Female	28	16	12	
Child-Pugh				0.719
A	38	18	20	
B	25	13	12	
Liver cirrhosis				0.124
yes	43	24	19	
no	20	7	13	
*Differentiation*				**0.014**
Well-moderate	23	16	7	
Poor	40	15	25	
*Tumor size*				**0.029**
≤5cm	36	22	14	
>5cm	27	9	18	
*Venous invasion*				**0.031**
Positive	37	14	23	
Negative	26	17	9	
*BCLC stage*				0.539
A	17	10	7	
B	33	16	17	
C	13	5	8	
*AFP level*				0.532
<400	33	15	18	
>400	30	16	14	

Notes: *P* < 0.05 by χ2 test. *P* < 0.05 was considered statistically significant.

Abbreviations: BCLC, Barcelona Clinic Liver Cancer; AFP, alpha-fetoprotein.

**Figure 3:**
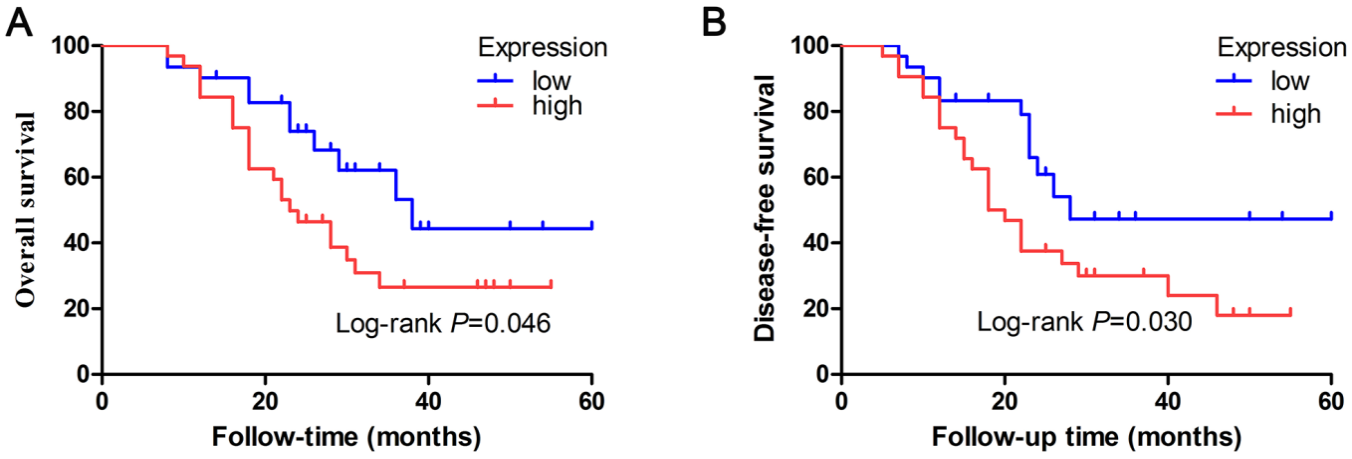
**Prognostic value of let-7e expression was evaluated by Kaplan–Meier survival analyses of HCC patients.** (**A**) HCC patients who had high let-7e expression had significantly worse OS rates compared with those who had low let-7e expression (*P=0.046). (**B**) Compared with HCC patients who had low let-7e expression, those who had high let-7e expression had significantly worse DFS rates (*P=*0.030).

To explore whether expression of let-7e was associated with prognosis in HCC patients, we used KM survival analyses to compare the overall survival (OS) and disease-free survival (DFS) rates between the high- and low-expression groups. Results revealed that median OS was 23 months in the high–let-7e expression group versus 38 months in the low–let-7e expression group. The high–let-7e expression group had a significantly worse OS rate than the low–let-7e expression group (*P=*0.046, log-rank test; Figure 3A). The median DFS rates of patients with high and low let-7e expression were 19 and 28 months, respectively. Compared with the low–let-7e expression group, the high–let-7e expression group had a significantly worse DFS rate (*P=*0.030, log-rank test; Figure 3B). Therefore, high let-7e expression was significantly associated with poor prognosis in HCC patients.

### Verification of let-7e expression in transfected HCC cells

**Figure 4:**
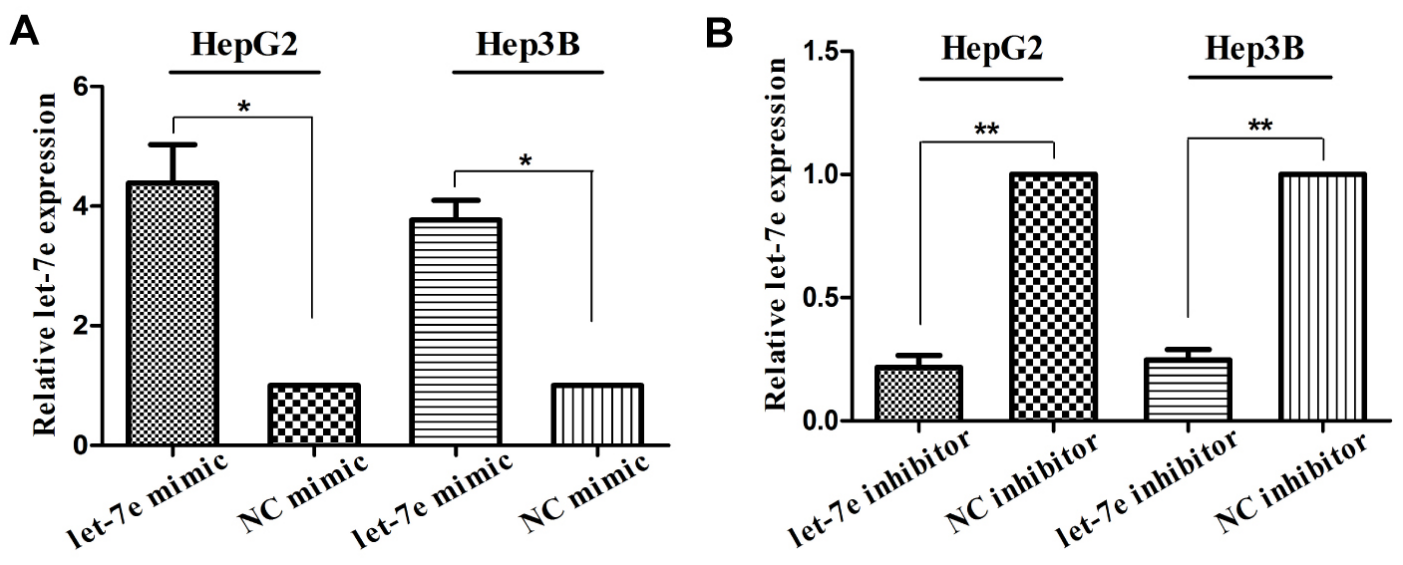
**Verification of let-7e expression in transfected HCC cells.** (**A**) Expression of let-7e was significantly upregulated in HepG2 and Hep3B HCC cells transfected with let-7e mimic compared with those transfected with control mimic. (**B**) Expression of let-7e was significantly downregulated in HepG2 and Hep3B HCC cells transfected with let-7e inhibitor compared with those transfected with control inhibitor (*P<0.05, **P<0.01, ***P<0.001). NC mimic–transfected and NC inhibitor-transfected cells were the control group in all experiments.

To investigate the BF of let-7e, we transfected let-7e mimic and let-7e inhibitor into HepG2 and Hep3B cell lines, respectively, to construct let-7e–expressing and let-7e–inhibited cell lines. We also transfected control mimic and inhibitor to construct NC cells for these two HCC cell lines. Before conducting experiments to determine the BF of let-7e, we confirmed the expression thereof in the transfected cell lines via RT-qPCR (Figure 4).

Expression of let-7e was significantly upregulated in HCC cells transfected with let-7e mimic than in those transfected with control mimic (Figure 4A). Conversely, let-7e expression was significantly downregulated in HCC cells transfected with let-7e inhibitor than in those transfected with control inhibitor (Figure 4B).

Using these transfected HCC cells, we conducted the following experiments to determine the BF of let-7e.

### Expression of let-7e affected the growth and proliferation of HCC cells

**Figure 5:**
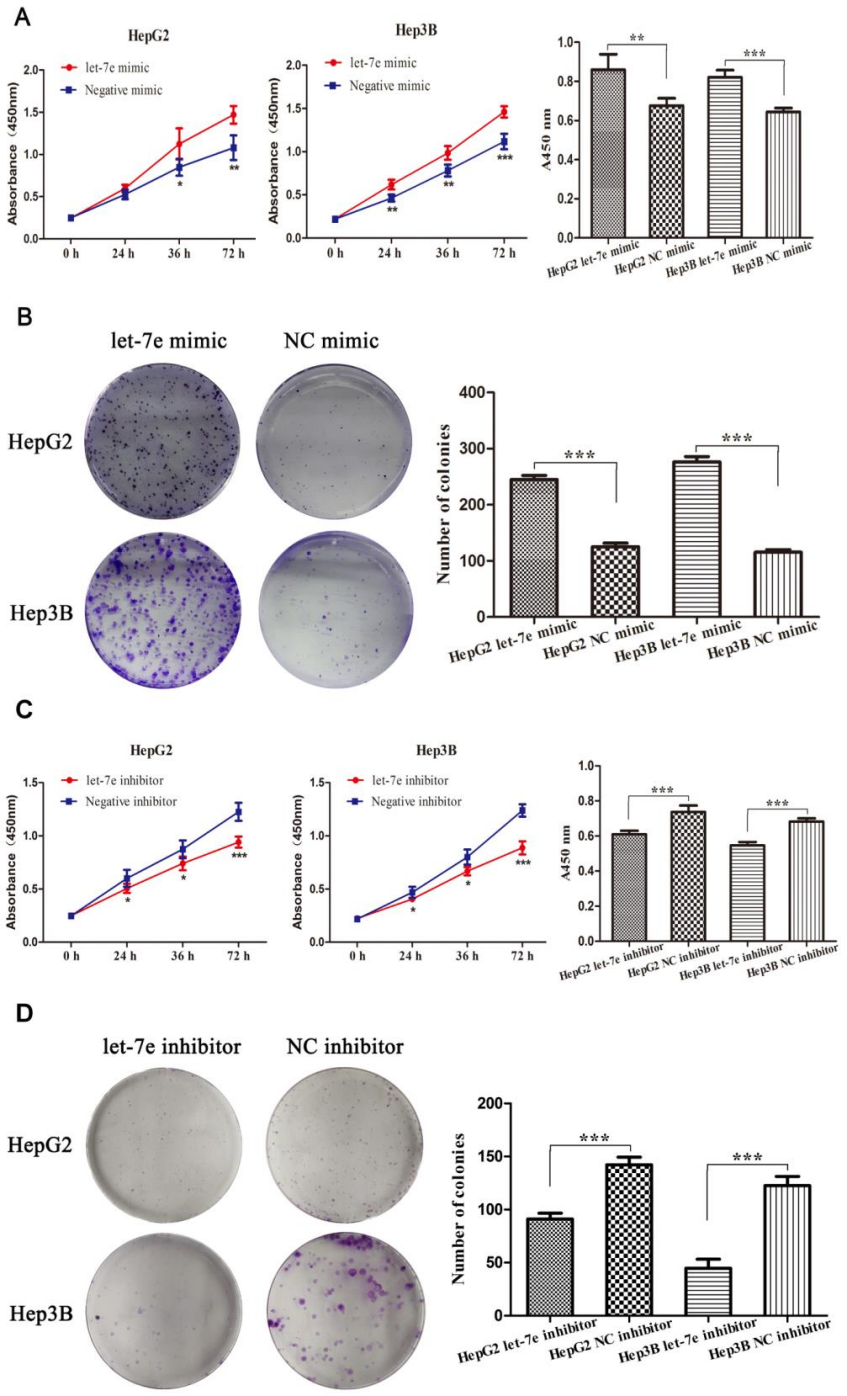
**Expression of let-7e affected the growth and proliferation of HCC cells.** (**A**) Results of cell viability assays showed that upregulated let-7e promoted the proliferation of HepG2 and Hep3B HCC cells. (**B**) Colony formation assays revealed that upregulated let-7e improved the colony formation efficiency of HepG2 and Hep3B HCC cells. (**C**) Downregulated let-7e inhibited the proliferation of HepG2 and Hep3B HCC cells. (**D**) Downregulated let-7e inhibited the colony formation efficiency of HepG2 and Hep3B HCC cells (*P<0.05, **P<0.01, ***P<0.001). NC mimic–transfected and NC inhibitor–transfected cells were the control group in all experiments.

To explore whether high let-7e expression affected the growth of HCC cells, we performed cell viability and colony formation assays in HepG2 and Hep3B HCC cells. Cell viability assay results showed that upregulated let-7e promoted the proliferation of HCC cells (Figure 5A), while the colony formation assays revealed that upregulated let-7e improved the cells’ colony formation efficiency (Figure 5B). To prove whether downregulated let-7e could inhibit the growth of HCC cells, we transfected HepG2 and Hep3B HCC cells with let-7e inhibitor and NC inhibitor and then performed cell viability and colony formation assays. The results showed that downregulated let-7e inhibited cell proliferation of HCC cells in cell viability assays (Figure 5C). The colony formation assays showed that downregulated let-7e inhibited the colony formation efficiency of these cells (Figure 5D).

### Expression of let-7e affected apoptosis of HCC cells

**Figure 6:**
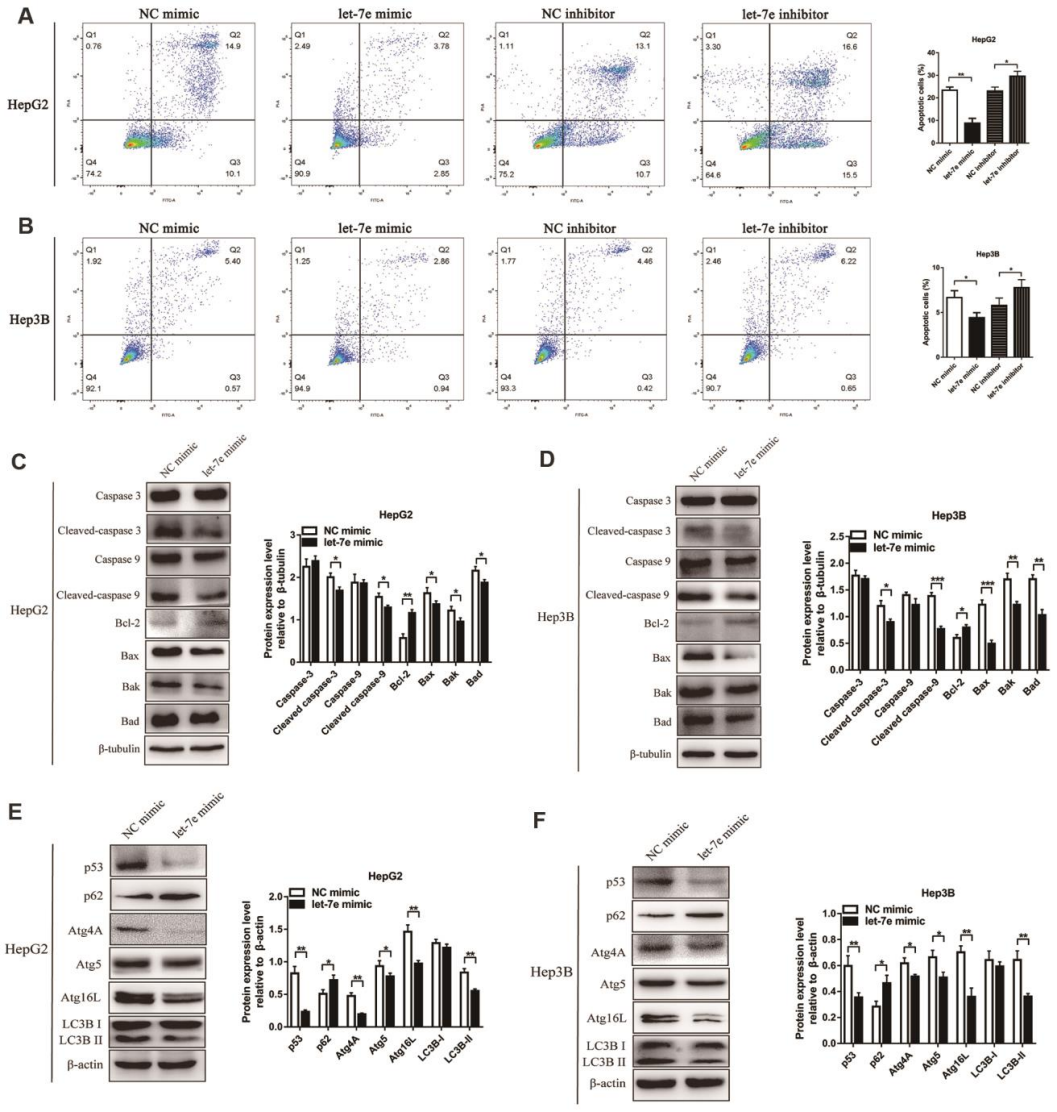
**Effects of let-7e expression on apoptosis and autophagy in HCC cells.** (**A**) Upregulated let-7e suppressed apoptosis of HepG2 cells, but downregulated let-7e initiated apoptosis in these cells. (**B**) Upregulated let-7e suppressed apoptosis of Hep3B cells, but downregulated let-7e initiated apoptosis in these cells. (**C**) WB analysis was used to detect the expression of several key cell apoptotic proteins in let-7e mimic–transfected HepG2 cells and NC mimic–transfected HepG2 cells. (**D**) Determination of the expression of several key cell apoptotic proteins in let-7e mimic–transfected Hep3B cells and NC mimic–transfected Hep3B cells. (**E**) Determination of the expression of key autophagy regulators in let-7e mimic–transfected HepG2 cells and NC mimic–transfected HepG2 cells. (**F**) Determination of the expression of key autophagy regulators in let-7e mimic–transfected Hep3B cells and NC mimic–transfected Hep3B cells (*P<0.05, **P<0.01, ***P<0.001). The NC mimic–transfected cells were as the control group in all experiments.

To explore whether let-7e’s promotion of HCC cell growth and proliferation was relevant to cell apoptosis, we analyzed apoptosis via flow cytometry (FCM). Results showed that the percentage of apoptotic cells was significantly lower in let-7e mimic–transfected cells but higher in let-7e inhibitor–transfected cells, which indicated that let-7e expression affected apoptosis in HCC cells (Figure 6A, 6B).

### Expression of let-7e affected apoptosis and autophagy of HCC cells

To further explore whether the functional mechanism of cell growth and proliferation affected by let-7e was related to mitochondrial apoptosis, we performed Western blot (WB) analysis to detect the expression of apoptosis-related proteins in the HCC cell lines. Results suggested that the levels of pro-apoptotic protein Bax, Bak, Bad, cleaved Caspase-9 and cleaved Caspase-3 were decreased, while that of the anti-apoptotic protein Bcl-2 was increased, in let-7e mimic–transfected HCC cells (Figure 6C, 6D).

Autophagy is a conserved cellular process considered to be associated with tumor progression, particularly in cell apoptosis [22, 23]. To explore the function of let-7e in autophagy of HCC cells, we detected the expression of autophagy-related proteins using WB analysis. The results suggested that expression levels of LC3, Atg4A, Atg5, Atg 16L and p53 were decreased but that of p62 was increased in let-7e mimic–transfected HCC cells (Figure 6E, 6F).

Taken together, our results indicated that let-7e might suppress cell apoptosis and autophagy via the p53 pathway, which induced the growth and proliferation of HCC cells.

## DISCUSSION

HCC is still one of the most malignant tumors, with increasing incidence and a high mortality rate [2, 24]. Though a variety of therapeutic methods have been developed, the recurrence and metastasis rates of HCC remain stubbornly high, leading to unsatisfactory and poor prognoses [25]. Because of the disease’s complicated pathogenesis and molecular mechanism [26, 27], finding an effective treatment is still a worldwide problem. Therefore, it is imperative to identify underlying molecular mechanisms and valuable biomarkers of HCC.

With the development of high-throughput technologies and bioinformatics analysis, omics sciences have been promoted over the last decade, and petabytes of molecular data on various human diseases have been collected [28]. This ever-growing amount of bioinformatics data contributes to our understanding of the physiopathological aspects of diverse diseases, including cancers [29]. In the present study, we aimed to identify novel miRNAs that could serve as biomarkers for diagnosis and prognosis in HCC, as well as to understand the MMs of HCC by analyzing and integrating several miRNA profiling datasets using several computational approaches. However, the huge quantity of data from the relevant databases is often not completely accurate, which generates conflicting results [21]. Therefore, after selecting the miRNAs most likely to be valuable from the results of our bioinformatics analyses, we explored their probable clinical values, BFs and MMs in HCC via analysis of clinical data and our own experiments.

MiRNAs, which are short, single-stranded ncRNAs 19–25 nucleotides long, are pivotal in the cellular processes of development, differentiation and aging [12–15]. In the current study, we first analyzed and integrated the GEO DataSets GSE6857, GSE22058 and GSE12264 to identify miRNA expression profiling datasets. By analyzing the differing expression levels of miRNA profiles, as well as performing ROC analyses of the GEO DataSets and survival analyses using the KM Plotter datasets, we identified three dysregulated miRNAs for further analyses: let-7b, let-7c and let-7e.

The let-7 family contains 13 members encoding nine mature miRNAs, including let-7a, let-7b, let-7c, let-7d, let-7e, let-7f, let-7g, let-7i and miR-98 [30]. The multiple functions of this family are extensively pleiotropic including oncogenic behavior; repression of oncogenes; and regulation of signaling pathways, cell cycle, apoptosis, epithelial–mesenchymal transition (EMT) and chemosensitivity in cells [31–57]. In previous studies, let-7b expression has been associated with prognosis in hepatoblastoma, HCC, melanoma and prostate cancer [32, 34, 58, 59]. Let-7c expression was related to the development of acute promyelocytic leukemia; HCC; and prostate, lung and endometrial cancers [34, 43, 60–63]. Other studies have found let-7e expression to be associated with melanoma; endometrial, prostate and ovarian cancers; and esophageal carcinoma [58, 59, 63–65]. The above referenced studies revealed that let-7b, let-7c and let-7e were multifunctional miRNAs involved in the development of various cancers. In the present study, we analyzed their most relevant neighboring and cross-linked genes using a functional-enrichment analysis tool. We also analyzed the top 10 relevant enriched TFs, which were ASCL2, EGR1, SP1, HOXA7, TCF3, MYC, NF1C, VSX2, SP4 and LHX3. These TFs participate in the occurrence and development of diverse cancers [66–77]. The selected miRNAs might play roles in HCC progression by influencing levels of these TFs. Cancer is regarded as a disease of communication between and within cells [78]. To explore how the selected miRNAs might affect progression of HCC via their BFs and via signaling pathways, we analyzed the target genes of the three miRNAs using GO and KEGG analyses. BF and pathway enrichment analysis results showed that these miRNAs regulated the phosphatidylinositol-4,5-bisphosphate 3-kinase (PI3K-)–protein kinase B (Akt signaling pathway), p53 and MAPK signaling pathways, all of which are considered classic signaling pathways involved in the development of cancers [78–80]. P53 is downstream protein affected by Akt, a central protein of the PI3K–Akt pathway; it mediates many signaling pathways in cancers and generates multifarious biological responses [81]. In addition, p53 can functionally interact with components of the MAPK signaling pathway, including p38 MAPK, extracellular-signal–related kinase (ERK) and stress-activated protein kinase (SAPK)–c-Jun N-terminal protein kinase (JNK) [82]. Therefore, we supposed that the selected miRNAs might play roles in HCC progression by affecting these interactional signaling pathways.

From the comprehensive results of above analyses, we supposed that let-7e was likely to play the most significant role in the diagnoses and prognoses of HCC patients. However, due to discrepancies in the relevant databases, bioinformatics data analyses often produce conflicting results [21]. Therefore, to identify the clinical value, BF and MM of let-7e, we based this study on our own data. First, we explored the association between let-7e expression and clinicopathological parameters. Our results suggested that high let-7e expression was significantly associated with poor tumor differentiation, larger tumor size and venous invasion. The results of KM survival analyses indicated that high let-7e expression was correlated with worse OS and DFS rates in HCC. Next, we performed cell viability and colony formation assays to identify the BF of let-7e in HCC cells. We found that upregulated let-7e promoted cell growth and colony formation efficiency in HCC cells, which indicated that this miRNA might act as an oncomiR.

MiRNAs promote cellular proliferation through mechanisms such as suppression of apoptosis and autophagy [83, 84]. In our study, we found that upregulated let-7e promoted cellular proliferation by suppressing apoptosis and autophagy in HCC cells. These are both highly conserved processes participating in cellular proliferation, death and homeostasis, and dysfunctions in either process can result in various human diseases [85, 86].

Apoptosis involves the extrinsic death receptor pathway and the intrinsic mitochondrial pathway [87]. The extrinsic pathway is activated by several death receptors, such as Fas Cluster of Differentiation 95 [CD95/Apo1),]/apolipoprotein 1 [APO1]), tumor necrosis factor receptors (TNFRs) and TNF-related apoptosis-inducing ligand receptors (TRAILRs). This in turn activates pro–Caspase-8, which then directly proteolytically cleaves and activates Caspase-3, resulting in mitochondrial damage [88]. The initiator caspase of the intrinsic apoptotic pathway is Caspase 9 [87]. Bcl-2 family proteins also tightly regulate this pathway. These proteins are comprised of pro-apoptotic members (such as Bax, Bak, Bad, BID, Bcl-Xs, Bim, Bik, HRK, Noxa and PUMA) and anti-apoptotic members (such as Bcl-2, Bcl-Xl, Bcl-W, Bfl-1 and MCL-1) [89]. In our studies, we revealed that let-7e could downregulate the pro-apoptotic protein Bax, Bad, Bad, cleaved Caspase-9 and cleaved Caspase-3 while upregulating the anti-apoptotic protein Bcl-2. Therefore, upregulation of let-7e suppressed apoptosis of HCC cells via the intrinsic mitochondrial pathway.

Autophagy usually occurs at the contact sites between the endoplasmic reticulum and mitochondria with the formation of the isolation membrane/phagophore. This membrane/phagophore contains macroproteins or even whole organelles which are sequestered into lysosomes for degradation [90]. Autophagy is a highly conserved process relying on the function of a core set of ATGs [91]. A series of signaling pathways initiate or regulate autophagy cascades, including the adenosine monophosphate–activated protein kinase (AMPK-)–mammalian target of rapamycin complex 1 (mTORC1), class I PI3K, Akt–mTOR, Ras–rapidly accelerated fibrosarcoma (Raf-1-)–mitogen-activated protein kinase 1/2 (MEK1/2-)–extracellular signal–regulated kinase 1/2 (ERK1/2,) and p53 signaling pathways [92]. Recent studies have found autophagy to be an upstream initiator of apoptosis and to regulate cell apoptosis by modulating Caspase and Bcl-2 family proteins [93]. Autophagy might be a guardian or executioner of apoptosis, depending on the surrounding micro-environment, therapeutic intervention and stage of carcinoma [93]. Therefore, in this study, we also detected the expression of autophagy-related proteins to explore the effect of let-7e on autophagy in HCC cells. We found that upregulated let-7e decreased the expression levels of LC3, Atg 4A, Atg5, Atg 16L and p53, but increased that of p62. From these results, we supposed that let-7e might suppress cell autophagy and apoptosis via the p53 signaling pathway, inducing the growth and proliferation of HCC cells.

In conclusion, let-7e was associated with poor prognosis in HCC patients and acted as an oncogene by suppressing autophagy and apoptosis in HCC cells, suggesting that it could be a novel biomarker for prognosis and target of treatment in HCC.

## MATERIALS AND METHODS

### Analysis of GEO databases

To identify miRNAs potentially involved in the development and progression of HCC, we reviewed the National Center for Biotechnology Information (NCBI; Bethesda, MD, USA) GEO databases. To find miRNA profiles significantly dysregulated in HCC, we analyzed three GEO DataSets: GSE6857, GSE22058 and GSE12264. Differences in miRNA expression between the tumor group (HCC) and normal group of the three datasets were compared using Student’s *t* test. *P* < 0.05 and fold change ≥ 1.5 were considered statistically significant. Then, we conducted Venn selections of differentially expressed miRNAs among the three lists using the online tool Venny version 2.1.0 (http://bioinfogp.cnb.csic.es/tools/venny/).

Heatmaps of differential miRNA expression between both groups in all three datasets were generated using the pheatmap package in R software (Ihaka and Gentleman, 1999). *P* < 0.05 was considered statistically significant.

### Diagnostic prediction of selected miRNAs

We evaluated the predictive power of the three selected miRNAs in HCC diagnosis using ROC curves. Clinical data were derived from GEO database GSE12264.

### Kaplan–Meier plotter

To evaluate the prognostic value of extracted-miRNA expression in liver cancers, we performed analyses using the KM Plotter. Cancer patients were divided into two groups, high and low miRNA expression, according to median values of miRNA expression; and KM survival curves were drawn in the plotter. *P* < 0.05 was considered statistically significant.

### Prediction of target genes of miRNAs

We analyzed the predictive power of target genes of the selected miRNAs and discovered the top 10 enriched TFs using a functional-enrichment analysis tool (FunRich). We also used FunRich to analyze the interaction network between the selected miRNAs and their target genes.

### Enrichment analysis of target genes

Using ClusterProfiler software version 3.11 (https://bioconductor.org/packages/release/bioc/html/clusterProfiler.html) [94, 95], we conducted functional-enrichment analysis of the predicted target genes of the selected miRNAs. GO analysis was conducted to describe functions of predicted genes, including BP, CC and MF. KEGG was used for pathway enrichment analysis based on significance at *P* < 0.05.

### Patients and tissue samples

We had earlier collected HCC tissues along with adjacent normal tissues at the Third Affiliated Hospital of Sun Yat-sen University (Guangzhou, China) after obtaining informed consent from all included patients. This study conformed to ethical and legal standards and was approved by the Research Ethics Committee of Sun Yat-sen University. Sixty-three tissue pairs, accompanied by patient clinical characteristics and other patient information, were included in this study. Enrollment criteria were as follows: histological diagnosis of HCC; no previous anti-cancer treatment before surgery; no distant metastasis; no other tumors found; and adequate clinical follow-up time (≥6 months). Clinicopathological parameters of included samples are briefly summarized in Table 2. In the first year after surgery, we followed up with patients every 3 months, then every 6 months over the subsequent several years. We assessed patients’ health status using blood routine tests, hepatic-function tests, serum alpha-fetoprotein (AFP) level tests, abdominal ultrasonography (US) and magnetic resonance imaging (MRI). Median follow-up time was 23 months (range, 8–60 months).

### Cell lines and culture

The human hepatocarcinoma cell lines in this study included HepG2 and Hep3B, which were both purchased from the Shanghai Cell Bank (Chinese Academy of Sciences, Shanghai, China). We cultured the cell lines in Dulbecco’s Modified Eagle’s Medium (DMEM) supplemented with 10% fetal bovine serum (FBS; GIBCO [Thermo Fisher Scientific], Grand Island, NY, USA) and put them in a humidified 5% CO_2_ incubator at 37° C.

### Real-time quantitative polymerase chain reaction (RT-qPCR)

We used TRIzol solution (Invitrogen; Corp., Carlsbad, CA, USA) to extract total RNA from HCC specimens and cell lines per manufacturer’s instructions. After determining concentrations of total RNA using a NanoDrop 2000 Spectrophotometer (Thermo Fisher Scientific, Waltham, MA, USA), we synthesized each complementary deoxyribonucleic acid (cDNA) sequence using a reverse transcriptase kit (Invitrogen); then, we used the cDNA as a template for RT-qPCR. Primer sequences of let-7b, let-7c, let-7e and U6 were devised and synthesized by Guangzhou Ribobio Co., Ltd. (Guangzhou, China). We used U6 small nucleolar RNA for normalization. Quantification of let-7b, let-7c and let-7e was performed using a Stem-Loop Real-Time PCR miRNA Kit (Ribobio). Primer sequences of ASCL2, EGR1, SP1, HOXA7, TCF3, MYC, NF1C, VSX2, SP4, LHX3 and glyceraldehyde 3-phosphate dehydrogenase (GAPDH) were devised and synthesized by Ribobio. GAPDH was used as an internal control. We determined relative expression of each gene via the 2^−ΔΔCt^ method.

### Cell transfection

We purchased let-7e mimic and let-7e inhibitor from Ribobio. After being cultured in a six-well culture dish for 24 h until the degree of cell fusion reached about ~70%, the HCC cell lines HepG2 and Hep3B were transfected with let-7e mimic or inhibitor and normal control (NC) mimic or inhibitor via transfection reagents (riboFECT CP Transfection Kit; Ribobio). The transfected concentration of mimic was 50 nmol, and that of inhibitor was 100 nmol.

### Cell viability assay

We used a Cell Counting Kit-8 (CCK-8; Dojindo Molecular Technologies, Inc., Kumamoto, Japan) to evaluate transfected cells’ viability. Cells were dispersed into and cultured in 96-well plates (2500 cells/well). To each well, we added 10μL CCK-8 reagent at the indicated time point. After incubating the transfected cells for 1 h at 37° C, we measured the absorbance of each well at 450 nm.

### Colony formation assay

Transfected HCC cell lines HepG2 and Hep3B were seeded into 6-well plates (1000 cells/well) for colony formation assays. Fourteen days later, we used 70% ethanol to immobilize the cell colonies for 15 min, after which we stained them for 10 min using 0.1% crystal violet. Finally, we counted the colonies and used this number to evaluate the transfected HCC cells’ colony formation capacity. Colony count was based on three different experiments and colonies in each well were manually counted three times.

### Cell apoptosis analysis

Cell apoptosis was measured using an Annexin V-FITC/PI apoptosis assay kit (Sangon Biotech, Shanghai, China). We analyzed the percentage of apoptosis on a CytoFlex flow cytometer (Beckman Coulter Life Sciences, Brea, CA, USA) per manufacturer’s instructions.

### Western blot

RIPA buffer (Pierce, Rockford, IL, USA) and BCA Protein Assay Kit (Pierce) were respectively used to extract proteins from cells and determine protein amounts. We separated the same amount of protein (20μg) via 6–12% SDS-PAGE. Then, the protein was transferred to PDVF membranes (MilliporeSigma, Burlington, MA, USA), which we blocked in 5% non-fat milk for 1 h. Next, we incubated the membranes overnight at 4° C with the following indicated primary antibodies, all of which were purchased from Cell Signaling Technology (CST; Danvers, MA, USA): Caspase-3 (1:1000; Cat. No. 9662), cleaved Caspase-3 (1:1000; Cat. No.9661), Caspase-9 (1:1000; Cat. No. 9502), cleaved Caspase-9 (1:1000; Cat. No. 9505), Bcl-2 (1:1000; Cat. No. 4223), Bax (1:1000; Cat. No. 2772), Bak (1:1000; Cat. No. 12105), Bad (1:1000; Cat. No. 9292), Bad (1:1000; Cat. No. 9292), β-Tubulin (1:1000; Cat. No. 2146), p53 (1:1000; Cat. No. 9282), p62 (1:1000; Cat. No. 39749), Atg4A (1:1000; Cat. No. 7613), Atg5 (1:1000; Cat. No. 12994), Atg16L (1:1000; Cat. No. 8089), LC3B (1:1000; Cat. No. 43566). The next day, membranes were incubated with HRP-labeled secondary antibody (1:2000) for 1 h, and a chemiluminescence kit (ECL) kit (KeyGen Biotech, Guangzhou, China) was used for visualization and detection of blots.

### Statistical analysis

The relationship between HCC patients’ clinicopathological characteristics and let-7e expression was analyzed via *χ*^2^ test. We used the KM method with a log-rank test for analysis of OS and DFS rates. All experimental data were analyzed using Student’s *t* test (two-sided). *P* < 0.05 was considered statistically significant. We used SPSS software version 22.0 (IBM, Armonk, NY, USA) for all statistical analyses.
